# Exploring Inclusion in Austria’s Breast Cancer Screening:A Dual-Perspective Study of Women with Intellectual Disabilities and Their Caregivers

**DOI:** 10.3390/ijerph23010124

**Published:** 2026-01-19

**Authors:** Theresa Wagner, Nourhan Makled, Katrina Scior, Laura Maria König, Matthias Unseld, Elisabeth Lucia Zeilinger

**Affiliations:** 1 Department of Clinical and Health Psychology, Faculty of Psychology, University of Vienna, 1010 Vienna, Austria; theresa.wagner@univie.ac.at (T.W.); laura.koenig@univie.ac.at (L.M.K.); 2 Vienna Doctoral School in Cognition, Behavior and Neuroscience, University of Vienna, 1010 Vienna, Austria; 3 Division of Palliative Medicine, Department of Medicine I, Medical University of Vienna, 1090 Vienna, Austria; 4 Research Department of Clinical, Educational and Health Psychology, UCL Division of Psychology and Language Sciences, London WC1E 7HB, UK; k.scior@ucl.ac.uk; 5 Department of Clinical Research SBG, Academy for Ageing Research, Haus der Barmherzigkeit, 1160 Vienna, Austria; matthias.unseld@hb.at; 6 Division of Health Psychology, Faculty of Psychology, Karl Landsteiner University of Health Sciences, 3500 Krems, Austria

**Keywords:** intellectual disabilities, learning disabilities, breast health awareness, health services, preventive care, qualitative research, health equity, cancer disparities, health care accessibility, person-centered care

## Abstract

**Highlights:**

**Public health relevance—How does this work relate to a public health issue?**

- Demonstrates systematic exclusion within an organized national breast cancer screening program, resulting in inequitable access for an underserved population—women with intellectual disabilities.
- Connects to broader public health priorities, including health equity, disability inclusion, and preventing avoidable late-stage diagnoses through early detection.

**Public health significance—Why is this work of significance to public health?**

- Identifies the complex interplay of structural, emotional, social, organizational, and attitudinal barriers, including diffusion of responsibility within the screening system, societal taboos, psychological factors, and the role of self-determination, that systematically limit screening participation.
- Generates actionable evidence to enhance program quality by incorporating accessibility and inclusion as core features of the screening rather than supplementary accommodations.

**Public health implications—What are the key implications or messages for practitioners, policy makers, and/or researchers in public health?**

- Underscores the need for person-centered healthcare, accessible communication and information, supportive environments, and screening pathways that accommodate diverse and individual needs within standard services.
- Calls for inclusion to be standardized through clear guidelines, robust data collection, mandatory ID-specific training, and clear accountability across the entire screening continuum.

**Abstract:**

Women with intellectual disabilities (IDs) face persistent health inequities, particularly in preventive services such as breast cancer screening, where participation rates remain disproportionately low. These disparities contribute to higher mortality and poorer survivorship outcomes, often linked to later-stage diagnoses. To better understand these challenges and inform the development of inclusive screening programs, this qualitative study conducted in Austria explored barriers, facilitators, and needs related to breast cancer screening from the dual perspectives of 17 women with mild-to-moderate IDs aged 45 and older and 10 caregivers. Semi-structured focus groups and interviews were analyzed thematically within a constructivist framework, integrating perspectives from both groups. Barriers included social taboos around sexuality, psychological distress, exclusion through standardized procedures, and unclear responsibility among stakeholders. Facilitators involved person-centered communication, accessible information, emotional and practical support, and familiar healthcare environments. Women with IDs expressed a strong desire for education, autonomy, and inclusion, while caregivers played a pivotal role in enabling access. These findings demonstrate that low screening participation among women with IDs is driven by systemic and organizational barriers rather than lack of health awareness or willingness to participate. Without structurally inclusive design, organized screening programs risk perpetuating preventable inequities in early detection. Embedding accessibility, clear accountability, and person-centered communication as standard features of breast cancer screening is therefore a public health priority to reduce avoidable late-stage diagnoses and narrow survival disparities for women with IDs.

Plain Language Summary

Women with intellectual disabilities are less likely to undergo breast cancer screening, even though they have a higher risk of dying from the disease compared to the general population [1]. This is unjust and shows a lack of health equity. This study aimed to explore barriers, facilitators, and needs related to breast cancer screening from the perspectives of women with intellectual disabilities and caregivers. We wanted to understand why women with intellectual disabilities take part in breast cancer screening less often and how access can be improved. The study was conducted in Austria. We talked to 17 women with mild to moderate intellectual disabilities and 10 caregivers about their experiences with breast cancer screening.

We found many barriers. Some come from the health system, like long waiting times, difficult forms, and machines that are not accessible. Others are emotional, like fear, pain, or bad past experiences. Stigma and taboos about women’s bodies also stop some from going. Caregivers can help women attend, but it is often unclear who is responsible for arranging this.

The women we spoke to care about their health. They wanted clear and easy information, and they wanted to make their own choices. Both women and caregivers had ideas for making screening easier, such as friendly staff, simple information, and accessible equipment.

We recommend changes to healthcare, training for staff, and more flexible screening programs. This would make breast cancer checks fair and accessible for all women.

## 1. Introduction

Women with intellectual disabilities (IDs) are systematically marginalized in healthcare, including preventive services such as breast cancer screening programs [2,3,4]. Compared to both men with ID and women without ID, they encounter more substantial barriers to accessing healthcare, often due to intersecting discrimination, structural disadvantages, and persistent social stigma [4].

Intellectual disabilities (IDs) are a group of etiologically diverse conditions characterized by an IQ below 70 and associated deficits in adaptive behavior as expressed in conceptual, social, and practical skills arising before the age of 18 years [5,6]. In the International Classifications of Diseases, 11th Revision (ICD-11), IDs are described as Disorders of Intellectual Development, with Code 6A00 distinguishing between mild, moderate, severe, and profound forms [7]. The global prevalence of ID is estimated at approximately 2% (female 1.37%) [8].

ID is associated with substantial health disparities reflected in the disproportionately high rates of cancer mortality among women with IDs [1,9]. A study from Canada identified a 2.28-fold higher risk of dying from breast cancer compared to women without ID [10]. Evidence also suggests that women with IDs experience poorer survivorship outcomes, primarily due to later-stage diagnoses and delays in care [11,12,13]. Despite these elevated risks, participation by women with IDs in the Netherlands’ organized breast cancer screening program was about 20%, significantly lower than that of women without IDs [14]. Supporting studies reported a 1.5-times-higher non-screening rate and a nearly fivefold increased risk of never being screened [9,14,15,16]. These disparities are further exacerbated by a higher comorbidity burden and social determinants of health, including high social vulnerability, belonging to a racial and ethnic minority, and geographic variations [17]. This highlights a significant gap in preventive care and reflects broader, multifaceted patterns of health inequity and exclusion from public health interventions. Reducing disparities in breast health awareness and screening is a critical determinant of survivorship, as earlier detection significantly improves treatment outcomes. Yet, for women with ID, barriers at the awareness, access, and follow-up stages create preventable delays in diagnosis and care [9,14,15].

Barriers to screening for women with ID include limited knowledge of breast cancer and screening procedures, consent issues, fear and anxiety, previous negative experiences, and the pivotal role of caregivers. Additionally, a lack of awareness and training among screening staff and caregivers further contributes to exclusionary practices, whereas the stated factors are often not gathered from women with ID themselves [9,18,19,20,21]. These barriers highlight that current breast cancer screening programs are not inclusively designed and that challenges exist at multiple levels. Any stage of the screening process is susceptible to inequalities, including the invitation process, access to screening, treatment, and outcomes, as screening should be viewed as a continuum of care [19,22].

Despite growing recognition of these challenges, there is still a lack of comprehensive evidence about the specific nature, timing, and mechanisms of these barriers, as well as a lack of effective strategies to overcome them. There is also limited engagement with how these barriers reflect broader socio-political determinants of health, including the ways in which health systems reproduce inequalities through their design and governance. Notably, most of the studies cited were conducted in the UK or the USA, reflecting healthcare systems that are not applicable to Austria, where the study was conducted. Furthermore, in Austria, there is no data on the incidence or prevalence of breast cancer or screening participation among women with ID. Standardized guidance, information guidelines, and toolkits on cancer screening for individuals with ID are lacking, as is training for health professionals on ID.

Furthermore, research from the perspective of women with ID is limited [9], leaving those directly affected without a voice. Most existing studies focus on either women with ID or their caregivers, with few considering both perspectives together [19]. However, bringing these perspectives into the same analytical frame allows for a richer understanding of the individual, relational, and structural dynamics that shape access to preventive care. The dual-perspective approach of the present study promotes knowledge and enriches the evidence base by offering a holistic, multifaceted, and more nuanced picture. Combining and linking separately collected data in a comprehensive analysis yields insights into the lived experiences of women with IDs and the systemic perspectives of their support networks. As most European countries, including Austria, operate organized free breast cancer screening programs, accessibility, equity, and inclusion should be ensured.

This study adopts a qualitative, dual-perspective approach to examine how women with ID and their caregivers experience and navigate breast cancer screening. The objective was to explore barriers, facilitators, and needs related to breast cancer screening from the dual perspectives of women with ID and their caregivers, to inform the development of inclusive screening programs. First-hand accounts from women with ID are particularly valuable for understanding their experiences, while caregivers, who play a critical role in supporting access to healthcare, offer complementary insights. The study ensures that the unique needs and perspectives of women with ID and caregivers are preserved by maintaining clarity on which subthemes originate from each group, allowing for the development of more targeted and effective interventions. This is one of the first studies to bring together the perspectives of women with ID and of caregivers in a European context with an organized national screening program.

## 2. Methods

### 2.1. Research Design

Semi-structured focus groups or interviews were conducted with women with ID aged 45 or older (*N* = 17), based on participant preferences. In parallel, caregivers (*N* = 10) of at least one woman with ID aged 45 or older were interviewed. To avoid bias, prevent inconsistent statements, and obtain a diverse range of views, the women with ID and the caregivers had no connection to each other. Information about recruitment is presented in Table 1, Domain 2, Participants Selection (Nr. 10–13). Distinct but complementary guidelines were developed to enable a comparative exploration of perspectives across both groups. All sessions were audio-recorded; transcribed verbatim, supported by the web-based transcription platform Trint; and analyzed using thematic analysis following the recommendations of Braun and Clarke [23,24]. None of the participants wanted the transcripts returned, and all agreed that the information provided could be used pseudonymously. The Supplementary Table S1 shows the topics covered and the material used. The guidelines for both samples covered the same topics, including knowledge about breast cancer (screening); experiences of breast cancer screening; barriers, facilitators, and needs for participation. The guideline for women with ID included a case study, while the caregiver guideline included additional questions about caring. Written informed consent was obtained from every participant prior to data collection. Informed consent procedures for women with ID were adapted to enhance accessibility, using easy-to-read language and materials, visual aids, additional time for explanations, and ongoing consent checks to confirm their willingness to participate. These adaptations were designed to carefully assess and support their capacity to consent. Comprehension was assessed using a brief teach-back approach, asking about the purpose of the study, procedures, and their rights. Only women who demonstrated adequate understanding proceeded to consent. Legal guardians were informed about the women’s participation via written correspondence (given to the women or their caregivers) or email, and they were given the right to object. In the event of objection, all data would have been deleted. No guardian objected. Legal guardians and caregivers were not involved during the data collection, except in two focus groups, where a caregiver was present but did not participate in the discussion (Table 1, Nr. 15). Data collection for the two samples was conducted separately between December 2022 and January 2024.

The study was pre-registered on the Open Science Framework (OSF; https://doi.org/10.17605/OSF.IO/A8ESK) and ethical approval was obtained from the Ethics Committee of the Medical University of Vienna (No. 1345/2022). The following sections describe the methods used for each participant group separately, beginning with the women with ID, followed by the caregivers. Table 1 presents the qualitative research reported in accordance with the Consolidated Criteria for Reporting Qualitative Research (COREQ [25]).

### 2.2. Interviews and Focus Groups with Women with ID

#### 2.2.1. Participants

The sample included 17 women with mild-to-moderate IDs, aged between 41 and 69 years (*M* = 56.9). Six participants took part in individual interviews, while the remaining 11 participated in four focus groups, each comprising two to three participants. The sample reflected a broad range of living arrangements and varying levels of support needs (see Table 2).

#### 2.2.2. Material

The focus groups and interviews followed a semi-structured qualitative format, incorporating open-ended questions, images, and a fictional case study featuring a woman with ID. To support communication, materials included easy-to-read language, pictures, and pictograms. The guide was developed by experts with psychological training and experience in ID, drawing on relevant literature and insights from pilot interviews [9,26,27,28,29]. The guide was reviewed and further refined with input from an experienced qualitative researcher with a medical background. A single guide was used for both individual interviews and focus groups, with minor adaptations tailored to each format.

#### 2.2.3. Procedure

Women with IDs were recruited using a maximum variation sampling approach to ensure diversity in experience and perspectives. Recruitment took place across both residential and community-based settings, including supported living and accommodation services, day centers, vocational programs, and self-advocacy organizations for people with ID, with study invitations disseminated via these organizations to eligible women and caregivers.

Recruitment was carried out via email to organizations providing services for people with different levels of ID, including accommodation providers, self-advocacy groups, and day centers. An invitation flyer designed in easy-to-read language at beginner (A1) to elementary (A2) level was used to ensure accessibility for individuals with ID. The invitation included information about the study topic, session duration, moderators, and contact details. Inclusion criteria were identifying as a woman aged 45 years or older, having an intellectual disability, and the ability to express oneself verbally. Participants’ socio-demographic data were collected at the beginning of the focus groups/interviews as part of establishing interpersonal relationships. Despite not meeting the age criterion, the data set of a 41-year-old individual was included due to her ongoing experience of mammography.

Sessions were conducted face-to-face in familiar environments, such as participants’ homes, workplaces, or residences, to ensure comfort and accessibility. Data was collected by three trained team members with psychological backgrounds. Sessions were attended only by participants and one moderator, with several exceptions. In three interviews and one focus group, an additional member of the research team was present. In two focus groups, a support person was also present but instructed to remain passive and not influence the discussion. The support persons themselves placed great importance on women with ID responding. They remained active in the background, doing other things, and repeatedly encouraged the women to respond independently when they were involved. Data from support persons was not included in the analysis. Sessions lasted between 45 and 65 min, while one interview lasted 30 min. Participants received a voucher as a thank-you. Recruitment ceased once the sample had been sufficiently reviewed for diversity and thematic saturation had been achieved. Thematic saturation was determined when no new themes emerged across two consecutive focus groups and interviews (*n* = 8), and existing codes seemed to sufficiently capture the diversity of perspectives. This decision was revisited and validated by the whole research team within the data acquisition process.

### 2.3. Interviews with Caregivers

#### 2.3.1. Participants

Ten caregivers (7 women, 3 men, 0 divers), aged between 29 and 62 years (*M* = 44.8 years), participated in the study. Two participants were family caregivers, while eight were caregivers by profession, with caregiving experience ranging from 2 to 30 years (*M* = 16.5 years). The women they cared for had a range of conditions, including varying forms of ID, Down’s syndrome, schizophrenia with minimal verbal communication, spastic paralysis, and motor and speech impairments (see Table 2).

#### 2.3.2. Material

Parallel to the guide of the women with ID, a semi-structured interview guide was developed by the study team based on existing literature [9,26,30,31] to explore barriers, facilitators, and needs influencing breast cancer screening participation. Experts in the field of IDs, qualitative research, and medicine revised it. The guide was pilot tested by a member of the research team to ensure clarity, structure, and suitability for the target group.

#### 2.3.3. Procedure

Caregivers were recruited through multiple channels to ensure diversity in caregiving experiences. Professional caregivers were approached via facilities supporting individuals with ID, while family caregivers were recruited through self-advocacy and peer support groups. Snowball sampling was also employed, with participants referring to other potential caregivers. Eligible participants were aged 18 years or older and were currently caring for, or had previously cared for, a woman with an ID aged over 45 who had experience with mammography.

Interviews were conducted in person at locations chosen by the participants, most commonly their workplace. Sessions were held in a quiet room with only the participant and one moderator of the research team present. Interviews lasted between 35 and 69 min. There was no incentive for participation. Recruitment concluded once the sample achieved sufficient diversity and thematic saturation. Thematic saturation was identified when no new themes emerged and the existing codes adequately represented diverse perspectives.

### 2.4. Data Analysis

Thematic analysis was employed with both data sets using an inductive, data-driven, latent, and constructivist approach. The study is grounded in a constructivist paradigm, which assumes that realities are co-constructed through social interaction (see Table 1). Three focus groups and four interviews of data from the women with ID, reporting a wide range of experiences, were coded jointly by three to four members of the coding team. The remaining focus group and two interviews were coded independently and then discussed in pairs to ensure consistency. To develop comprehensive coding schemes for the caregiver interviews, two transcripts were initially coded independently by two researchers, followed by a discussion to refine the coding. This process was repeated for a further two interviews. The remaining six interviews were then coded independently and reviewed by a single researcher. Following the reorganization, discussion, and clarification of the coding scheme for both data sets, the final coding schemes were applied to all focus groups and interviews respectively. Once the codes were clustered, potential themes were discussed by the coding team, the lead researcher, and an experienced qualitative researcher. The themes were reviewed, defined, and named. After both data sets were analyzed independently, the emerged themes were clustered, and common themes were identified, as the identified themes and sub-themes covered the same content or could be assigned to an overarching theme. MAXQDA (2022.5) software was used to aid in the analyses. The coding team consisted of researchers with a psychological background and trained in qualitative methods. Reflexive practices were regularly implemented to minimize bias, resolve disagreements and ensure that the analysis was conducted systematically, critically, transparently, and sensitively with respect to the data and participants. These practices included regular team debriefs involving an additional researcher unfamiliar with the data; collaborative discussion and coding; revisiting transcripts and auditing decisions made; documentation of assumptions; and active reflection to become aware of one’s own biases. Additionally, the lead researcher was consulted regularly and provided an experienced and critical perspective.

## 3. Results

**Figure 1:**
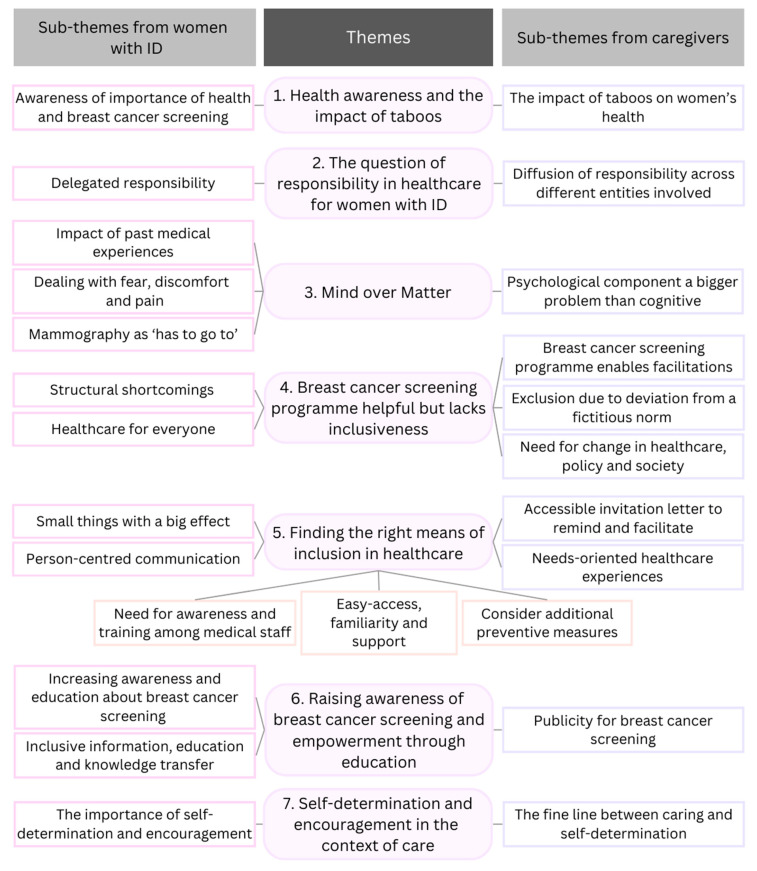
Themes and sub-themes from focus groups and interviews with women with intellectual disabilities (ID) and caregivers. The Figure shows the themes and sub-themes derived from thematic analysis of interviews and focus groups with 27 participants (17 women with intellectual disabilities and 10 caregivers). The sub-themes on the left represent the women with IDs and those on the right represent the caregivers. Theme five comprises three combined sub-themes, incorporating codes from both groups.

Seven common themes were identified from focus groups and interviews with women with IDs and interviews with caregivers, covering the dual-perspective analysis. Each theme includes at least one sub-theme that emerged from each sample group: in Figure 1, those on the left represent the women with IDs, and those on the right reflect the caregivers. Theme five comprises three combined sub-themes, including codes from both groups. For ease of attribution, quotes are labeled as ‘P_ID_’ for participants with ID and ‘P_C_’ for caregivers. As a woman with ID (P_ID_05) dropped out, the number of participants ‘P_ID_’ goes up to 18.

All verbatim quotations are additionally compiled in Supplementary Table S2, organized by themes and sub-themes.

*Theme 1: Health awareness and the impact of taboos*

This theme captures the interplay between health awareness and social taboos in the healthcare of women with ID. Women with IDs showed a strong *awareness of the importance of health and breast cancer screening.* They valued the screening for early detection and expressed a generally positive attitude towards it, describing it as necessary to maintain health: “You have to make sure you stay healthy somehow” (P_ID_04). Despite acknowledging the discomfort of mammography, participants expressed relief that it exists: “I think that if I’m standing there and it’s the final stage, I’d prefer to know early” (P_ID_07), and many would advise others to participate: “Definitely have a mammogram, because that’s prevention so that she (woman of the fictional case study) doesn’t get cancer. That’s what I’d advise her to do” (P_ID_18).

In contrast, caregivers highlighted how societal taboos around sexuality and intimacy negatively impact the health of women with IDs, captured in the sub-theme *the impact of taboos on women’s health.* These were linked to the perception that health checks like gynecological visits or breast cancer screening are unnecessary for women with IDs, as described here: “Unfortunately, it’s so, that many people feel that many things are unnecessary for disabled people” (P_C_08). This belief led some families to refuse preventive care, as reflected in expressions such as “We have all grown old without it” (P_C_01). In the care context, the intimate nature of breast cancer prevention, including mammography and breast palpation, was described as a complicated and sensitive issue, as reported: “That can also be interpreted as grabbing” (P_C_08), and “A sensitive topic. It’s a difficult topic” (P_C_01).

*Theme 2: The question of responsibility in healthcare for women with IDs*

Many women with ID were unaware of the existing organized breast cancer screening program. One participant stated: “I don’t know where to turn” (P_ID_14). Some recalled receiving invitation letters, while others did not. The organization of the screening participation varied widely from person to person, often depending on the initiative and recommendations of caregivers, gynecologists, or family members, “I do that (mammography) with Mum. When she goes, I go” (P_ID_18). The sub-theme *delegated responsibility* reflects this experience described by the women with ID. The sub-theme *diffusion of responsibility across different entities involved* emerged from the interviews with caregivers. Multiple actors, such as gynecologists, caregivers, facility management, legal representatives, and family members, were involved in the healthcare of women with IDs. Caregivers described relying on gynecologists’ recommendations and referrals for screening, as explained: “That this (mammography) is initiated by the gynecologist, that is trusted” (P_C_06), while medical decisions are generally made by legal guardians. This resulted in inconsistent screening participation, with no single person taking full responsibility. Caregivers also noted the challenge of managing prevention as an additional task, like “Preventive things tend to be seen as a burden” (P_C_06), which requires extra energy and coordination within their daily workload, especially when facing staff shortages or challenging behavior of clients. One person described the following: “Preventive examinations require extra effort and stress, particularly for the person themselves. Due to the effort involved, these examinations take a back seat, and we always have to consider how they might affect the person’s mood and well-being” (P_C_10). They explained that screening participation often depended on personal attitudes towards healthcare: “I think it depends entirely on the respective caregivers to be engaged, to ensure the breast cancer screening is integrated” (P_C_07).

*Theme 3: Mind over Matter*

This theme encompasses the dynamics of psychological, emotional, and experiential factors influencing screening participation, framing *mammography as ‘has to go to’*, with one woman describing, “I always say: ‘Close your eyes and go for it.’” (P_ID_17). Although the importance of mammography was widely recognized, women are *dealing with fear, discomfort, and pain,* acting as barriers. Fear and anxiety were recurring themes, including fear of doctors, fear of bad results, nervousness, and intense tension, as one person described: “I was a bit nervous” (P_ID_15). While some women reported no pain or discomfort, others described the mammography as uncomfortable and painful, with pain sometimes linked to breast size. *Impact of past medical experiences* also emerged from the interviews and focus groups with women with IDs influencing mammography participation, as stated: “You have to proceed with caution. Many women have had bad experiences” (P_ID_01).

Caregivers emphasize in the sub-theme *psychological component a bigger problem than cognitive* that psychological factors often posed greater challenge than cognitive limitations. Fear and discomfort were named as significant barriers, with caregivers observing nervousness, stress, worry about what would happen, agitation, and even panic during appointments. In extreme cases, women exhibited strong reactions, such as crying, screaming, running away, or refusing to sit still, “Some are very nervous, running up and down, shouting and swearing because they don’t want to wait, etc.” (P_C_03). One caregiver described: “It (mammography) is a challenge for women with ID, which is why we are there. We can help them weigh up the consequences. So, for them, it’s basically a mandatory appointment, which can be anything from annoying to exhausting” (P_C_05). Caregivers reported that women’s anxiety was regulated through distraction techniques, such as singing or using their phones. Both groups emphasized that previous positive or negative experiences with medical staff strongly shaped willingness to return.

*Theme 4: Breast cancer screening program helpful but lacking inclusiveness*

Women with IDs described *structural shortcomings* such as long waiting times, financial barriers, excessive paperwork, and not being taken seriously by medical staff. “They (women with ID) are hindered by the feeling that they are not being taken seriously or that no time is being given to them” (P_ID_01). They expressed a desire for *healthcare for everyone,* tailored to their needs: “People should be more accommodating towards those with special needs. (…) It requires more training to learn that you can’t get dressed so quickly or anything like that” (P_ID_07). Suggestions included having multiple doctors at the same place, more specialized doctors, more specialized providers, home visits (e.g., mobile breast cancer screening units), and ID-specific services. Many felt they had little influence over the healthcare system.

Caregivers generally viewed the screening program as well organized and facilitating regular care. However, *the screening program enables facilitation*; the sub-theme *exclusion due to deviation from a fictitious norm* underlines that it is neither inclusive nor flexible. Women who deviated from a fictitious norm due to cognitive, physical, or behavioral differences were systematically excluded. For example, mammography requires standing, and the lack of technical adaptations means that women with physical disabilities are excluded, as explained: “This device was designed for people who can stand normally, and we actually had to stop the mammogram” (P_C_06). Caregivers noted that intersecting factors like physical disabilities, high body weight, affective disorders, autism, or blindness often led to inadequate treatment or dismissal by healthcare providers, who were unprepared to adapt their practices. Non-verbal clients or those with sensory sensitivities were particularly vulnerable to exclusion.

Caregivers echoed the *need for change in healthcare, policy, and society* to make healthcare inclusive. They advocated for political action and standardized guidelines, such as routine annual health checks for women with ID and simplified processes. Caregivers advocated for broader societal changes, such as recognizing the individuality of people with ID and addressing stigma and discrimination, as caregivers explained: “Society is not prepared for our clients” (P_C_01) and “It is a question for society, whether this is relevant, and whether doctors feel compelled or not” (P_C_05).

*Theme 5: Finding the right means of inclusion in healthcare*

This theme summarizes ideas and suggestions that can foster greater inclusion of women with ID in healthcare. The sub-theme *small things with a big effect* included suggestions like bringing a comfort item (e.g., a cuddly toy), providing emotional support (e.g., handholding, hugs), or rewards (e.g., snacks). One woman further explained that “I distracted myself with music” (P_ID_08), which led to a discussion about the music type, with opinions ranging from “heavy metal would be better” (P_ID_09) to “so classical music” (P_ID_08). These things helped reduce anxiety and made medical visits more manageable. *Person-centered communication,* as the second sub-theme, was identified as the basis of inclusive healthcare, with women with IDs favoring clear, respectful explanations tailored to their individual preferences (e.g., receiving results).

Caregivers highlighted the value of invitation letters as reminders to encourage regular participation. However, they found the current format unsuitable due to inaccessible language and content. They suggested using easy-to-read language, pictograms or pictures, along with flyers explaining the procedure in an accessible manner.

*Needs-oriented healthcare experiences* capture the idea of caregivers to create a more approachable environment for women with ID. Drawing analogies to child-friendly approaches, they suggested using tools like picture books and visual aids to educate women. Practical strategies, such as avoiding the use of white doctors’ coats (still commonly worn by doctors in Austria), incorporating distractions (e.g., videos or music), and offering rewards or treats like sweets, were mentioned.

Both women with IDs and caregivers emphasized *the need for awareness and training among medical staff* and the critical role of medical staff in creating an inclusive healthcare system. Women valued friendliness, patience, and being addressed directly: “He [doctor] was always talking to the support person, which got on my nerves” (P_ID_07). Interactions that included humor, responsiveness, and willingness to adapt to individual needs were experienced as beneficial.

Many women with ID and caregivers reported negative experiences. Doctors were described as dismissive, unprepared, or hesitant to work with them: “First of all, the medical practice was not barrier-free. Then we somehow managed to get the wheelchair in and he [doctor] said ‘no, he doesn’t treat (woman with ID)’” (P_C_08). Caregivers observed that some providers seemed overwhelmed or unwilling to treat women with IDs. Participants strongly advocated for mandatory training and inclusion of ID topics in medical curricula.

The next sub-theme, *easy access, familiarity, and support,* which was identified in both groups, highlights the importance of barrier-free environments, short waiting times, and accessible locations. Familiar doctors, procedures, and settings helped build trust and reduce fear. Women appreciated the presence of support persons during visits, “If possible, parents should be there. If not, then the primary caregiver. Someone I trust” (P_ID_07).

The last sub-theme, *consider additional preventive measures,* captures that breast ultrasound and palpation might be options if mammography is not possible. Breast ultrasound was seen as a feasible alternative or complement to mammography. While some women with ID reported positive experiences with breast palpation, others initially found it strange and uncomfortable. Caregivers noted that self-palpation was often not performed regularly, with many women with IDs and caregivers preferring this to was performed by professionals. Education on self-exams was recommended.

*Theme 6: Raising awareness of breast cancer screening and empowerment through education*

Women with IDs expressed in the sub-theme *increasing awareness and education about breast cancer screening* a strong desire for more education and information about breast cancer, prevention and screening procedures: “I think there needs to be much more education” (P_ID_07) and “That the doctor talked to me. That was good for me. Because I didn’t know anything about it (mammograms) before, is it something bad, is it not something bad?” (P_ID_04). Lack of understanding increased discomfort. The importance of proactive and clear communication to be able to make informed decisions was stated.

The sub-theme *inclusive information, education, and knowledge transfer* emphasizes the need for accessible education methods. Many women with IDs noted a lack of easy-to-read information and difficulties understanding formal or technical language. Slow, patient, and calm explanations were described as helpful, as were visual aids like pictures, videos, or even dolls to demonstrate procedures. Women with IDs suggested, “a book in easy-to-read language with pictures” (P_ID_07) and using information booklets or media.

Caregivers also advocated for *publicity for breast cancer screening.* They emphasized the importance of public outreach and normalizing breast cancer screening to increase awareness and participation, as suggested: “Making the topic visible. With good slogans that stick in people’s minds, it becomes visible and is no longer a taboo subject. That it simply becomes a topic in society. You could even make it a friend’s activity and go for a breast screening together (laughing). Then you can go out for coffee and cake afterwards” (P_C_03).

Within residential facilities, screening and palpation were rarely discussed unless a diagnosis occurred. Caregivers recommended consistent education within facilities, using pictograms, videos, and easy-to-read materials, along with public campaigns (e.g., posters and TV advertisements). They also stressed addressing stigma and fear associated with cancer through public discussions.

*Theme 7: Self-determination and encouragement in the context of care*

Women with ID expressed a desire for autonomy in healthcare decisions: “This is my body. I decide what happens. I can be reminded. But I decide if I go” (P_ID_01). Encouragement with emphasizing the harmlessness of caregivers, friends, or peers was described as particularly helpful in overcoming fear or hesitation. Hearing about others’ experiences with mammography or talking to a friend helped some women make decisions about their own participation.

Caregivers reflected on *the fine line between caring and self-determination*. Many supported an approach of guidance without coercion, tailored to each woman’s needs. However, they acknowledged limits: “The will of the patient in my sister’s case would be no examination at all, ever in her life. (…) But if you always keep the will, where do we go?” (P_C_09). They emphasized that this fine line between caregiving and self-determination requires careful navigation.

## 4. Discussion

This dual-perspective study identified a complex interplay of structural, emotional, and social barriers that hinder access to breast cancer screening for women with ID. Through the perspectives of both women with ID and their caregivers, our findings highlight systemic shortcomings, fragmented responsibilities, and a persistent lack of inclusive healthcare design and delivery. Key themes included the impact of societal taboos, unclear responsibility structures, psychological barriers, a lack of inclusive design and delivery in healthcare services, and the importance of self-determination.

Notably, the identified barriers are not the result of individual factors but rather reflect systemic failures. The findings underscore that the current Austrian breast cancer screening program for women between 45 and 74 years, although nationally organized and free of charge, is not inclusively designed. Women who deviate from a presumed “norm” due to cognitive, physical, or behavioral differences are frequently excluded. This exclusion reflects broader structural discrimination.

Negative past experiences within the healthcare system, such as being dismissed, misunderstood, or excluded due to a lack of accessibility, can undermine trust and create emotional barriers. The fear, anxiety, and confusion associated with mammography, as reported by women with ID and their caregivers, indicate that the psychological burden is just as significant as the logistical challenges. Fear and negative provider attitudes have been identified as major deterrents to participation in national cancer screening programs, including those for breast, cervical, and colorectal cancer [9,18,30,32]. Similar patterns have been observed in other clinical contexts, such as palliative care, where healthcare professionals report limited preparedness, emotional strain, and systemic constraints when caring for vulnerable patient groups [33]. Stress and anxiety before, during, and after screening, including fear of pain, diagnosis, and unfamiliar environments, have been identified as consistent barriers across breast and cervical screening programs [34,35]. Evidence from cervical screening programs suggests that person-centered education, preparatory visits, and accessible information can reduce distress and encourage participation, highlighting the importance of inclusive screening design [19,21,30,34,35].

Discrimination and stigmatization are further reflected in taboos around the sexuality of women with ID, as revealed in interviews with caregivers. Sexuality and intimacy remain taboo topics, even within the context of preventive healthcare. Societal attitudes are imposed on women with IDs from the outside, contributing to misconceptions about the necessity of health checks for them and undermining their access to health prevention. Other studies on family caregivers’ perspectives also reported stigma, including discomfort with sexuality and doubts about the necessity of exams [36,37].

Interestingly, this topic only arose in interviews with caregivers. The women with ID themselves demonstrated a strong health awareness and a positive attitude towards breast cancer prevention, consistent with the findings of another study interviewing women with ID about receiving mammography [38]. Embarrassment and shame have been described as barriers to breast cancer screening by women with ID themselves [38,39]. However, in our study, the women did not experience embarrassment or shame, suggesting that such taboos may originate externally and act as an indirect mechanism for excluding women with ID from health care. The present study’s dual-perspective analysis reveals a tension and conflicting interaction between health awareness and societal taboos. Women with ID, despite being aware of the importance of screening, may face significant barriers when societal taboos limit their access to support and information. By avoiding discussions about intimate health topics, such as mammograms or breast self-examination, whether due to limited awareness or protective intent, as noted in a recent scoping review [40], caregivers may reinforce these taboos, further hindering access to essential healthcare equally important for all women.

Building on this pattern, prior studies described low cancer knowledge, including limited awareness of risk factors and symptoms, among paid/unpaid caregivers, family members, and healthcare professionals [18,40,41,42]. This study further supports that insufficient awareness and training among screening staff and caregivers contributed to exclusionary practices. Together, these findings point to the importance of addressing not only educational and training shortcomings but also broader societal and policy-level changes to ensure adequate cancer care and equitable access. Recent systematic evidence also highlights that, while cancer screening can provide important benefits, the absence of outcome evidence for adults with ID, along with documented risks relating to test validity, consent, and psychological burden, underscores the need for carefully adapted, inclusive approaches and rigorous evaluation of benefit-harm trade-offs in this population [22].

Although an organized breast screening program exists in Austria, the diffusion of responsibility across caregivers, legal guardians, and professionals results in inconsistent access. The absence of clear decision-making processes can delay or prevent participation by limiting follow-through. This challenge is similarly reported in scoping reviews describing uncertainty over responsibility for facilitating cancer awareness and the absence of practice guidelines across all stages of the breast cancer care pathway [40,43].

Ensuring equitable healthcare accessibility and inclusivity requires tackling a variety of factors. Recommendations from women with ID and caregivers include adopting person-centered communication, making small accommodations like bringing a favorite object, and providing easy, barrier-free access. Familiarity and trust were identified as crucial factors in encouraging participation, in line with prior research on colorectal cancer screening, indicating that these interpersonal aspects enhance participation [32]. Regular breast self-examination and ultrasound, both described as feasible in this study, should not be overlooked as additional screening options. Among individuals with severe motor and intellectual disabilities, ultrasonography has been reported as an effective and feasible tool for breast cancer screening [44].

While caregivers proposed drawing on child-friendly approaches, this raises ethical concerns. Infantilization risks undermining autonomy and dignity [45]. Instead, health care must strike a balance between support and self-determination, recognizing individuals with ID as adults with rights and preferences.

To ensure self-determination and autonomy of women with ID, support and healthcare services should be designed to empower and encourage them. Empowerment through education was strongly emphasized in the data, with calls for greater attention to breast cancer screening. The women with IDs explicitly expressed a strong desire to receive information in accessible formats, identifying this as a key enabling factor. The findings align with other studies showing that women with ID lack awareness about breast cancer warning signs, risk factors, and the breast screening program [38,39]. Tailored health information could strengthen the health literacy of both women with ID and their caregivers [41].

The findings of this study are consistent with evidence from other cancer screening programs for women with ID, particularly with regard to cervical cancer screening. Across screening contexts, limited access to appropriate education has been shown to increase anxiety and restrict informed participation. This is often reinforced by assumptions about women’s ability to engage in preventive care [19,30,34,36]. Intimate procedures involving physical touch and bodily exposure are also reported as distressing when explanations, consent processes, and supportive communication are inadequate, with discomfort primarily shaped by contextual factors such as trust, familiarity, and societal taboos rather than the procedure itself [21,30,35].

### 4.1. Implications

The results underscore the critical need to systematically recognize and include women with ID in the planning, design, and delivery of preventive healthcare services. The complex, multi-stakeholder nature of their healthcare requires coordinated, multilevel strategies to ensure equitable access. A key implication is the need to challenge persistent societal stigma and taboos, especially those relating to sexuality and bodily autonomy of women with ID. These cultural narratives restrict access to preventive care and reinforce exclusion. Public health messaging must actively portray women with ID as autonomous individuals entitled to the same health rights as others. Equally important is to actively include women with ID to challenge stereotypes and discrimination. Programs that enable individuals with ID to advocate for themselves are essential for reducing stigma and fostering their inclusion in society and healthcare [46,47].

Education is key to empowerment. Providing accessible, tailored health information using easy-to-read materials, pictograms, visual aids, videos, and peer-led sessions can improve understanding and reduce fear. Rather than focusing solely on breast cancer, promoting ‘breast awareness’ more broadly may be less threatening and more empowering [48]. To raise overall awareness of breast screening, information should be publicized more widely through TV adverts, posters, and engaging slogans, ensuring information is accessible to everyone. However, women with ID are a highly diverse group with significant variations in cognitive abilities, communication skills, prior healthcare experiences, and anxiety related to screening. Information provision should always be tailored at the individual level rather than implemented through universal guidance, whether it is provided directly to the woman or via a caregiver or legal guardian.

Given the limited awareness and knowledge of cancer and cancer risk factors among caregivers, family members, and medical staff, they should be equipped with the necessary knowledge and skills. Healthcare professionals need mandatory ID-specific training about their roles and responsibilities to ensure they can provide adequate and effective care for individuals with ID. The framework of the disability-competent care (DCC) model, promoting a person-centered care approach, along with disability-competent communication training (e.g., speaking clearly and directly to the women with ID) and guideline-based adjustments, should be integrated into medical curricula [49]. This kind of training enables flexible, needs-led care and ensures that women with different abilities can participate meaningfully in preventive health services.

Adopting person-centered approaches that create a more comfortable and less stressful experience is essential for reducing psychological barriers, such as fear and anxiety. Supportive and non-hierarchical environments should be fostered during medical visits to facilitate interaction on an equal footing, value the needs of the individual, use clear and accessible communication, and provide accommodations. Offering reasonable adjustments by healthcare providers, such as extended/multiple appointment times, a pre-visit of mammography facilities, and other personalized support (e.g., positioning aids such as pillows and reviewing the environment for triggers such as lighting), would make the process more accessible. Devices or adjustments that allow screening while seated are further crucial. While examples of good practices regarding reasonable adjustments and accessible mammography protocols exist [50,51,52], there is a need to develop standardized protocols to ensure clinical governance. Such protocols could serve as an international standard.

Guidelines and accountability structures are needed to ensure that breast cancer screening is not overlooked due to diffusion of responsibility, negative attitudes, or a lack of understanding. Standardized, transnational government policies, guidelines, and processes must clearly define responsibilities and ensure that interdisciplinary healthcare decisions supported by these policies are made in the best interests of individuals with ID. Further policies such as providing financial or social incentives can ensure consistent participation. Importantly, such protocols and guidelines should serve only as frameworks to ensure the inclusion of people with ID in healthcare, while recognizing and allowing for person-centered care, individual needs, and preferences, which must remain central throughout the entire screening process.

A system of legal guardianship for making healthcare decisions on behalf of people with IDs, as exists in Austria, should be critically discussed as a matter of urgency. Participation should not depend on the workload or attitudes of one individual support person or legal guardian. As a recent review reported, healthcare decision-making for adults with ID is often marked by exclusion and assumptions of incapacity, calling for further research on supported decision-making [53]. Since professional and family caregivers play a critical role in initiating healthcare for people with ID, their work should be valued through fair wages and support mechanisms.

A key step towards person-centered care and the development of targeted programs is the collection of registry data. Systematic data analysis could help identify regions with low participation rates. Implementing tailored interventions, such as mobile screening units in underserved areas, could enable equal healthcare access. Additionally, it would provide individualized, needs-based support not only for women with ID.

Ultimately, breast cancer screening for women with ID must not remain an individual burden. Equity in healthcare depends on inclusive systems that redistribute responsibility and provide active support. It is only through empowerment, accessibility, and structural reform that women with ID can fully and independently exercise their right to health. Healthcare systems should be held accountable for establishing the necessary inclusive frameworks. Delays in screening contribute to later-stage diagnoses and poorer survival rates. By removing structural and informational barriers, the strategies we propose have the potential to narrow the survival gap between women with and without ID. Tailored awareness campaigns, inclusive communication, and accessible screening options are therefore not only preventive measures but also critical components of survivorship care.

### 4.2. Limitations

While this study offers valuable insights into the barriers and facilitators of breast cancer screening for women with ID, several limitations must be acknowledged. Although women with ID were included in the study, it was not conducted as participatory research. They were only involved in data collection and did not participate in study design or analysis. Future research should adopt co-creative or participatory methods to ensure the full inclusion of people with ID in all stages of the research process, as demonstrated in recent equity-focused health studies, e.g., [54]. Additionally, the study included only women with mild to moderate ID. Women with severe or profound ID, who often face even greater access barriers, were not directly represented. The caregivers included were not the direct caregivers of the same women with ID who participated. However, caregivers of women across the spectrum of disability severity were included, providing insights into these groups’ specific challenges. While this design allowed us to capture a range of experiences, it may also have introduced some disparities. Future research could benefit from a paired design, interviewing women and their respective caregivers together, to explore dyadic dynamics more directly. The presence of support persons during some interviews may have influenced responses through social desirability bias. Although moderators were trained to facilitate participant autonomy, subtle power dynamics may still have shaped some accounts.

Furthermore, the sample lacked diversity in terms of ethnic, cultural, and socioeconomic backgrounds. All participants were recruited within Austria, providing rich, context-specific insights that may resonate with similar healthcare systems, though the study does not aim for generalization. Intersectional dimensions, such as migration status, language barriers, or poverty, were not explicitly addressed but may further exacerbate inequalities [17]. While caregivers provided rich data, family caregivers were underrepresented in the sample. Their perspectives may differ significantly from those of professional caregivers, especially in regard to decision-making authority and emotional involvement.

## 5. Conclusions

This study demonstrates the substantial and systemic barriers that women with intellectual disabilities (ID) face in accessing breast cancer screening, despite the existence of organized national programs. The findings reveal that exclusion is not merely the result of individual limitations but is driven by a combination of structural inaccessibility, societal stigma, emotional and psychological challenges, and a lack of clear responsibility within the healthcare system.

To address these challenges, a shift towards inclusive, person-centered healthcare is essential. This requires clear communication, emotionally supportive environments, accessible information, and systems designed to accommodate diverse needs. Structural changes are also needed to establish accountability, equip healthcare providers with the necessary skills and sensitivity to serve this population, and ensure that participation in preventive care is not dependent on chance or goodwill.

Ultimately, improving access to breast cancer screening for women with ID is both an equity imperative and a survivorship strategy. Addressing disparities in awareness, screening participation, and access to healthcare will help ensure that all women, regardless of their disability status, can benefit equally from advances in breast cancer prevention, early detection, and treatment. Future efforts to close this gap must be guided by empowerment, inclusion, and systemic reform, ensuring that preventive healthcare is accessible to all.

## Figures and Tables

**Table 1:** Using COREQ checklist for reporting qualitative research.

Domain 1: Research Team and Reflexivity		
Personal characteristics		
1. Interviewer/facilitator	Which author/s conducted the interview or focus group?	Regarding the interviews and focus groups with women with IDs, TW conducted four interviews and three focus groups. One interview and one focus group were conducted by MK. NB conducted one interview. An additional person was present at three of the interviews (AF, SK) and one focus group (LH). NM conducted the interviews with the caregivers.
2. Credentials	What were the researcher’s credentials? E.g., PhD, MD	TW was an MSc in psychology and a PhD student. AF, SK, MK, NB, and LH were BSc in psychology. NM was a medical student.
3. Occupation	What was their occupation at the time of the study?	TW, AF, SK, MK, NB, LH, and NM were research fellows. SK, MK, and LH were interns, while the others were employed team members. NM conducted her thesis within the project.
4. Gender	Was the researcher male or female?	TW, AF, MK, NB, NM, and LH were female, and SK was male.
5. Experience and training	What experience or training did the researcher have?	All had training in qualitative research methodologies. TW, NB, and NM had experience with qualitative methods, including facilitating focus groups and conducting semi-structured interviews. All received training about ID.
Relationship with participants		
6. Relationship established	Was a relationship established prior to study commencement?	No prior relationship was established between the researchers and participants prior to obtaining informed consent.
7. Participant’s knowledge of the interviewer	What did the participants know about the researcher? E.g., personal goals, reasons for performing the research	Participants knew where the researchers worked, about the research project, and the purpose of the research.
8. Interviewer characteristics	What characteristics were reported about the interviewer/facilitator? E.g., Bias, assumptions, reasons, and interests in the research topic	The professional background of the interviewer was disclosed. Participants were informed that the study was conducted as part of a research project at the University of Vienna. No assumptions were made regarding the research topic, participants, or expected outcomes. The interviewer’s interest in the research topic stemmed from its relevance to their academic work and equitable access to healthcare.
Domain 2: Study design		
Theoretical framework		
9. Methodological orientation and Theory	What methodological orientation was stated to underpin the study? E.g., grounded theory, discourse analysis, ethnography, phenomenology, content analysis	A phenomenological approach situated within a constructivist paradigm was adopted to explore and understand individuals’ lived experiences, which emphasizes participants’ own constructions and descriptions, and treats knowledge as co-constructed within social contexts. Data were analyzed using reflexive Thematic Analysis (TA) with an inductive, data-driven, latent, and constructivist approach.
Participants selection		
10. Sampling	How were participants selected? E.g., purposive, convenience, consecutive, snowball	We employed purposive maximum-variation sampling to recruit women with ID and caregivers, ensuring diversity in characteristics, experiences, and perspectives. Participants were recruited via snowball sampling and continued until thematic saturation was reached.
11. Method of approach	How were participants approached? E.g., face-to-face, telephone, mail, email	Women with ID were recruited through invitation flyers via email distributed through various organizations providing residential and community-based services, including supported living facilities, vocational programs, self-advocacy organizations, and day centers for people with different levels of ID. Caregivers were recruited through facilities supporting individuals with ID, while family caregivers were recruited through self-advocacy and peer support groups.
12. Sample size	How many participants were in the study?	17 women with ID and 10 caregivers participated in the study.
13. Non-participation	How many people refused to participate or dropped out? Reasons?	Due to the recruitment strategy, refusals by individuals could not be systematically tracked. One woman with ID withdrew after contacting us and arranging an interview appointment. Upon arrival at the facility, she declined to proceed with the interview. A caregiver noted she was having a difficult day.
Setting		
14. Setting of data collection	Where was the data collected? E.g., home, clinic, workplace	Data were collected face-to-face in familiar environments for the women with IDs, such as participants’ homes, workplaces, or residences. Interviews with the caregivers were conducted face-to-face at locations chosen by the participants, mostly at their workplace or at the workplace (practice) of the interviewer.
15. Presence of non-participants	Was anyone else present besides the participants and researchers?	In two focus groups, a support person was present but instructed to remain passive and not influence the discussion.
16. Description of sample	What are the important characteristics of the sample? E.g., demographic data, date	Women with ID: aged 45 years or older, having an intellectual disability, ability to express oneself verbally. Caregivers: aged 18 years or older, currently or previously cared for a woman with ID aged over 45 years who had experience with mammography.
Data collection		
17. Interview guide	Were questions, prompts, and guides provided by the authors? Was it pilot tested?	Semi-structured guidelines were used. For the focus groups and interviews with the women with IDs, a booklet with some questions accompanied by images, voting, and pictograms was handed out. The interview guideline of the caregivers was pilot-tested and also used as a template for the focus groups and interviews with the women with IDs.
18. Repeat interviews	Were repeat interviews carried out? If yes, how many?	No repeated interviews were carried out.
19. Audio/visual recording	Did the research use audio or visual recording to collect the data?	The focus groups and interviews were audio-recorded.
20. Field notes	Were field notes made during and/or after the interview or focus group?	Field notes were made during some focus groups and interviews to clarify unclear statements. The notes were not used for transcription or analysis.
21. Duration	What was the duration of the interviews or focus group?	Focus groups and interviews with the women with IDs lasted between 45 and 65 min, whereas one interview lasted 30 min. Interviews with the caregiver lasted between 35 and 69 min.
22. Data saturation	Was data saturation discussed?	Data saturation was discussed within the research team and the PI (ELZ). Recruitment was stopped once the samples had been reviewed to ensure sufficient diversity and thematic saturation.
23. Transcripts returned	Were transcripts returned to participants for comment and/or correction?	None of the participants requested the return of the transcripts.
Domain 3: Analysis and findings		
Data analysis		
24. Number of data coders	How many data coders coded the data?	Data coding process for the focus groups and interviews with the women with IDs involved four members of the research team (TW, AF, AH, MH). Data that emerged from the caregivers were coded by two members of the research team (TW, MH). A third perspective was given by NM.
25. Description of the coding tree	Did the authors provide a description of the coding tree?	A description of how the coding tree was created is provided (Section 2.4.). However, due to the richness of the coding trees of both data sets, the coding trees themselves are not included.
26. Derivation of themes	Were themes identified in advance or derived from the data?	Themes were derived from the data during analysis.
27. Software	What software, if applicable, was used to manage the data?	The transcription process was facilitated by the software Trint. MAXQDA (2022.5) was used to support the analyses.
28. Participant checking	Did participants provide feedback on the findings?	No, the participants did not provide feedback on the findings.
Reporting		
29. Quotations presented	Were participant quotations presented to illustrate the themes/findings? Was each quotation identified? E.g., participant number	In the Section 3, participant quotations were represented to illustrate the themes. Each quotation can be assigned to its respective sample and participant number.
30. Data and findings consistent	Was there consistency between the data presented and the findings?	All findings were derived from the data. The data presented and the findings were consistent.
31. Clarity of major themes	Were major themes clearly presented in the findings?	Yes, major themes were clearly presented in the Section 3.
32. Clarity of minor themes	Is there a description of diverse cases or a discussion of minor themes?	The minor subthemes are described alongside the major themes (Figure 1) and illustrated by quotations in the Section 3.

**Table 2:** Characteristics of women with IDs and caregivers.

Characteristic of Women with IDs *	*N* = 17		
Interviews (P_ID_01–07)	6		
Focus groups (P_ID_08–18) **	11		
Focus group 1 Focus group 2 Focus group 3 Focus group 4	3 3 2 3		
	Range (years)	***M***	***SD***
Age	41–69	56.9	7.5
		*n*	%
Gender	Women	17	100
Living Area	Urban	17	100
Living Situation	With Family	5	29.4
	Alone/with Partner	2	11.8
	Assisted (24/7 support)	2	11.8
	Semi-Assisted (hourly support)	1	5.9
	Missing	7	41.2
Legal Guardianship ***	Yes	14	82.4
	No	3	17.6
**Characteristic of caregivers**	***N =* 10**		
	Range (years)	*M*	*SD*
Age	29–62	44.8	11.3
Work experience (*n* = 8)	2–30	16.5	10.2
		*n*	%
Gender	Women	7	70
	Men	3	30
	Divers ****	0	0
Caregiver	Professional	8	80
	Family	2	20
Conditions mentioned of women they care for	All forms of ID, schizophrenia, spastic paralysis, motor and speech disorders, Down’s syndrome		

* The screening history of the women with ID could not be provided adequately due to recall issues. ** As one woman with an ID (P_ID_05) dropped out, participant coding extends up to P_ID_18. *** Under Austrian law, people with IDs often have someone making decisions and acting on their behalf. **** Gender category acknowledging people who do not exclusively identify as male or female.

## Data Availability

The data supporting the findings of this study are not publicly available due to ethical and privacy considerations, as they contain information that could compromise the confidentiality of research participants.

## Supplementary Materials

The following supporting information can be downloaded at: https://www.mdpi.com/article/10.3390/ijerph23010124/s1, Table S1: Topics covered in the focus groups and interviews with material used; Table S2: Themes and subthemes with quotations.
