# Supplementary material for: Exploring Inclusion in Austria’s Breast Cancer Screening:A Dual-Perspective Study of Women with Intellectual Disabilities and Their Caregivers
Source: Int J Environ Res Public Health. 2026 Jan 19;23(1):124. doi: 10.3390/ijerph23010124 (PMC12840729; doi:10.3390/ijerph23010124)
Supplement: Supplementary file 1 [file ijerph-23-00124-s001.zip › ijerph-3987094-supplementary.pdf]

Exploring Inclusion in Austria's Breast Cancer Screening:
A Dual-Perspective Study of Women with Intellectual Disabilities and their Caregivers

Supplementary Material

Table S1: Topics covered in the focus groups and interviews with material used

Focus groups/interviews with women with ID	Interviews with caregivers
**1. Welcome, informed consent and demographic data**<br>**Material:** Flipchart with timeline, presentation, sheets, pictures, pictograms, smilies, voting	**1. Welcome, informed consent and demographic data**
**2. Questions about health and doctor visits; Knowledge of cancer, breast cancer, associations with breast cancer and early detection** (breast palpation, breast ultrasound, mammography)<br>E.g.: Do you know what cancer/breast cancer is? What do you associate with breast cancer? Do you know how to detect breast cancer early?<br>Explanation about cancer, breast cancer, and early detection<br>**Material:** Presentation, pictures, pictograms, materials form 'Simply talking about health and illness' and METACOM	**2. Knowledge of, attitude towards, and experience with the existing breast cancer screening program**<br>E.g.: Have you had any experience with the Austrian breast cancer screening program?
**3. Attitude towards early detection; Information about breast cancer/ early detection wanted and how information should be presented**<br>E.g.: What is your opinion on early detection tests? Would you like to know more about preventive examinations?<br>**Material:** Presentation, pictures, voting	**3. Organizing healthcare and prevention for women with ID in facility/in family**<br>E.g.: How is preventive healthcare generally organized? Who is responsible for what? Who decides which examinations are done?
**4. Participation, experience with breast cancer early detection (breast palpation, breast ultrasound, mammography); Feelings during these examinations**<br>E.g.: Have you ever had a breast screening examination? What was it like for you? How did you feel during the breast examination?<br>**Material:** Presentation, pictures, pictograms, voting, smilies	**4. Experience of breast cancer screening among supported women; Participation; Barriers and facilitators; Factors for non-participation**<br>E.g.: Do you have experience of supporting women with ID through breast cancer screenings? If participation, which factors encourage regular participation? If no participation, which factors cause non-participation?
**5. Experience with screening program (invitation letter, organization); Barriers and facilitators of breast cancer screening program**	**5. Support needs to participate**<br>E.g.: What would be helpful for woman with ID/caregiver/ medical staff?

E.g.: What was your experience of the breast examination? What was the process like? Were there any problems? What was helpful?<br>**Material:** Presentation, pictures, pictograms, voting, smilies	
**6. Support needs to participate in breast cancer screening program**<br>E.g.: What would you need?<br>**Material:** Presentation, pictograms, pictures	**6. Specific questions about caregiving: Responsibility for health/prevention and own feelings**<br>E.g.: How do you feel about being (jointly) responsible for the health and care of another person?
**7. If no experience with early detection/ recall problems**<br>- Questions about willingness to perform breast palpation, breast ultrasound, mammogram<br>- Barriers, facilitators, emotions, needs<br>E.g.: Would you have a mammogram? Why yes/no? What stops you from having a mammogram?	
**8. Case Study: Sandra does not know whether to get a mammogram**<br>- Description of Sandra, 50-year-old women with intellectual disabilities<br>E.g.: What advice would you give to Sandra?<br>**Material:** Pictures, pictograms	
**9. Summary and conclusion**<br>Summary of topics, positive conclusion, current wellbeing<br>**Material:** Pictures, pictograms, smilies, voting	

Note. All interviews and focus groups were conducted in German. For the focus groups and interviews with women with ID, all questions and materials were designed using easy-to-read language (language level A1-A2). Participants' current state of well-being was consistently assessed during the focus groups and interviews. The materials used were taken from the Monique brochure by the ONCODEFI Association, which is available in German (https://www.lebenshilfe.de/informieren/familie/krebsinformationen).

Supplementary Material

Table S2: Themes and subthemes with quotations

The table summarizes the themes and sub-themes, along with quotations, from the thematic analysis of interviews and focus groups with 27 participants (17 women with intellectual disabilities and 10 caregivers). The sub-themes with quotations on the left reflect perspectives of the women with intellectual disabilities (PID), while those on the right represent the caregivers' perspectives (PC). Theme five consists of three merged sub-themes.

Theme 1: Health awareness and the impact of taboos	
Awareness of importance of health and breast cancer screening<br>• "You have to make sure you stay healthy somehow" (PID04).<br>• "I think that if I'm standing there and it's the final stage, I'd prefer to know early" (PID07).<br>• "Definitely have a mammogram, because that's prevention so that she (woman of the fictional case study) doesn't get cancer. That's what I'd advise her to do" (PID18).	*The impact of taboos on women's health*<br>• "Unfortunately, it's so, that many people feel that many things are unnecessary for disabled people" (PC08).<br>• "We have all grown old without it" (PC01).<br>• "That can also be interpreted as grabbing" (PC08).<br>• "A sensitive topic. It's a difficult topic" (PC01).
Theme 2: The question of responsibility in healthcare for women with ID	
Delegated responsibility<br>• "I don't know where to turn" (PID14).<br>• "I do that (mammography) with Mum. When she goes, I go" (PID18).	*Diffusion of responsibility across different entities involved*<br>• "That this (mammography) is initiated by the gynecologist, that is trusted" (PC06).<br>• "Preventive things tend to be seen as a burden" (PC06).<br>• "Preventive examinations require extra effort and stress, particularly for the person themselves. Due to the effort involved, these examinations take a back seat, and we always have to consider how they might affect the person's mood and well-being" (PC10).<br>• "I think it depends entirely on the respective caregivers to be engaged, to ensure the breast cancer screening is integrated" (PC07).
Theme 3: Mind over Matter	

Mammography as 'has to go to'<br>• "I always say: 'Close your eyes and go for it.' " (PID17).	*Psychological component a bigger problem than cognitive*<br>• "Some are very nervous, running up and down, shouting and swearing because they don't want to wait, etc." (PC03)".<br>• "It (mammography) is a challenge for women with ID, which is why we are there. We can help them weigh up the consequences. So, for them, it's basically a mandatory appointment, which can be anything from annoying to exhausting" (PC05).
Dealing with fear, discomfort and pain<br>• "I was a bit nervous" (PID15).	
Impact of past medical experiences<br>• "You have to proceed with caution. Many women have had bad experiences" (PID01).	
Theme 4: Breast cancer screening program helpful but lacking inclusiveness	
Structural shortcomings<br>• They (women with ID) are hindered by the feeling that they are not being taken seriously or that no time is being given to them" (PID01).	*Breast cancer screening program enables facilitation*
Healthcare for everyone<br>• "People should be more accommodating towards those with special needs. (…) It requires more training to learn that you can't get dressed so quickly or anything like that" (PID07).	*Exclusion due to deviation from a fictitious norm*<br>• "This device was designed for people who can stand normally, and we actually had to stop the mammogram" (PC06).
	Need for change in healthcare, policy and society<br>• "Society is not prepared for our clients" (PC01).<br>• "It is a question for society, whether this is relevant, and whether doctors feel compelled or not" (PC05).
Theme 5: Finding the right means of inclusion in healthcare	

<table>
<tr><td>Small things with a big effect
"I distracted myself with music" ($P_{ID}08$).
"heavy metal would be better" ($P_{ID}09$).
"so classical music" ($P_{ID}08$).</td><td>Accessible invitation letter to remind and facilitate</td></tr>
<tr><td>Person-centred communication</td><td>Needs-oriented healthcare experiences</td></tr>
<tr><td colspan="2">Need for awareness and training among medical staff</td></tr>
<tr><td>"He [doctor] was always talking to the support person, which got on my nerves" ($P_{ID}07$).</td><td>"First of all, the medical practice was not barrier-free. Then we somehow managed to get the wheelchair in and he [doctor] said 'no, he doesn't treat (woman with ID)' " ($P_{C}08$).</td></tr>
<tr><td colspan="2">Easy access, familiarity and support</td></tr>
<tr><td>"If possible, parents should be there. If not, then the primary caregiver. Someone I trust" ($P_{ID}07$).</td><td></td></tr>
<tr><td colspan="2">Consider additional preventive measures</td></tr>
<tr><td colspan="2">Theme 6: Raising awareness of breast cancer screening and empowerment through education</td></tr>
<tr><td>Increasing awareness and education about breast cancer screening
"I think there needs to be much more education" ($P_{ID}07$).
"That the doctor talked to me. That was good for me. Because I didn't know anything about it (mammograms) before, is it something bad, is it not something bad?" ($P_{ID}04$).</td><td>Publicity for breast cancer screening
"Making the topic visible. With good slogans that stick in people's minds, it becomes visible and is no longer a taboo subject. That it simply becomes a topic in society. You could even make it a friend's activity and go for a breast screening together (laughing). Then you can go out for coffee and cake afterwards" ($P_{C}03$).</td></tr>
<tr><td>Inclusive information, education and knowledge transfer
"a book in easy-to-read language with pictures" ($P_{ID}07$).</td><td></td></tr>
<tr><td colspan="2">Theme 7: Self-determination and encouragement in the context of care</td></tr>
<tr><td>The importance of self-determination and encouragement
"This is my body. I decide what happens. I can be reminded. But I decide if I go" ($P_{ID}01$).</td><td>The fine line between caring and self-determination
The will of the patient in my sister's case would be no examination at all, ever in her life. (…) But if you always keep the will, where do we go?" ($P_{C}09$)</td></tr>
</table>
